# Taxonomic reassessment and rejection of the spider family Fonteferreidae Wunderlich, 2023 (Araneae)

**DOI:** 10.3897/zookeys.1278.181771

**Published:** 2026-04-27

**Authors:** Lara Lopardo, Nadine Dupérré, Gustavo Hormiga, Peter Michalik

**Affiliations:** 1 Zoological Institute and Museum, University of Greifswald, Loitzer Str. 26, 17489 Greifswald, Germany Zoological Institute and Museum, University of Greifswald Greifswald Germany; 2 Museum of Nature Hamburg, Zoologie, Leibniz-Institute for the Analysis of Biodiversity Change (LIB), Center for Taxonomy and Morphology, Martin-Luther-King-Platz 3, 20146 Hamburg, Germany Department of Biological Sciences, The George Washington University Washington United States of America; 3 Department of Biological Sciences, The George Washington University, 2029 G St. NW, Washington, DC 20052, USA Museum of Nature Hamburg, Zoologie, Leibniz-Institute for the Analysis of Biodiversity Change Hamburg Germany

**Keywords:** Araneoidea, *nomina dubia*, symphytognathoids

## Abstract

The taxonomic validity of the spider family Fonteferreidae Wunderlich, 2023 is re-evaluated in light of new morphological evidence. This araneoid family was originally established based on a single specimen, the male holotype, that lacked both distinctive somatic characters and reproductive structures. Upon re-examination, the holotype specimen was found to be a subadult male, and consequently the diagnostic traits previously cited to justify its familial status are found to be unsubstantiated. In absence of the necessary empirical evidence, the erection of the family Fonteferreidae, the genus *Fonteferrea* Wunderlich, 2023, and *F. minutissima* Wunderlich, 2023 must be rejected. We recommend that *Fonteferrea minutissima*, the genus *Fonteferrea*, and the family Fonteferreidae be formally treated as a ***nomina dubia*** within Araneae.

*“Extraordinary claims require extraordinary evidence”*

Carl Sagan (1979)

## Introduction

In a recent publication, Wunderlich (2023) described a new extant araneoid family, Fonteferreidae Wunderlich, 2023 (originally misspelled as Fonteferridae), based on a single specimen collected in the Algarve region of Portugal. Hypothesizing new spider families is not rare. During the last two decades the number of recognized extant spider families has increased from 114 to 139 (WSC 2025). Nearly all these additions stem from reinterpretations of previously known morphologies informed by new phylogenetic hypotheses (e.g. Montana et al. 2025; Morris et al. 2025). Only in a few cases the new families have been erected to accommodate entirely new discoveries evidently lacking any clear affinities to known families, such as Trogloraptoridae (Griswold et al. 2012) and Myrmecicultoridae (Ramírez et al. 2019). The discovery of a purportedly new spider family in Europe, a region that has been extensively studied over the past centuries, is both intriguing and extraordinary.

Accurate species-level identification of spiders (Araneae) relies heavily on diagnostic morphological features, particularly those associated with reproductive structures of adults. At the family level, classifications are generally supported by robust phylogenetic hypotheses and clearly defined synapomorphies (e.g. Ramírez 2014). The recently established monotypic family Fonteferreidae challenges these taxonomic necessities since its original description. The family was diagnosed based on a single male specimen and a set of vague, non-diagnostic morphological traits, including “prosoma raised”, “clypeus short”, “opisthosoma quite high, higher than long”, the presence of “8 eyes”, an imprecise distribution of “bristles” on the legs, “legs distinctly annulated”, and a “body length 0.75 mm” (Wunderlich 2023: 16). Neither in isolation nor in combination these putative diagnostic somatic features suggest a new or an existing familial circumscription. On the other hand, the diagnostic features of the single male specimen included some unusual characters: “bulbus quite simple (*), bearing few hairs (!) (**), without protruding sclerites but a small ‘fleshy’ structure, embolus rather short”. The simplicity of the palpal bulb was further characterized by “strongly reduced structures/sclerites of the bulbus (and embolus)”. Wunderlich also noted that the presence of “hairs” in the bulb (the cymbium typically bears setae), is a “quite rare character”, that has been previously observed in other spider families, particularly the extant species *Enoplognatha minuscula* Wunderlich, 2023 (Theridiidae). Unfortunately, he noted, the details of “the exact insertion, the fine structure and the function of these hairs are still unknown” (Wunderlich 2023: 16). In fact, the identification of “hairs” on the spider bulb would constitute a highly significant discovery, warranting further investigation. This is particularly pertinent given that the prevailing paradigm in arachnology asserts the absence of sensilla in this context (e.g. Dederichs et al. 2019).

In this paper, we present a critical reassessment of Fonteferreidae, with particular focus on the male palpal reproductive structures of the single known specimen. After studying the holotype of *Fonteferrea minutissima* we cannot find any convincing evidence supporting the conjecture that the specimen belongs to a new spider family as originally argued. We further demonstrate the absence of setae on the male bulbs in at least the two extant species where this feature was originally reported. We argue that the presence of setae in the palpal bulb is not a reliable diagnostic character but instead likely results from observational error. Lastly, we emphasize that, in spiders, the use of adult specimens is critical for accurately hypothesizing taxonomic groups at any rank.

## Materials and methods

We examined the holotype of *Fonteferrea minutissima* Wunderlich, 2023 and *Enoplognatha minuscula* Wunderlich, 2023, both housed at the Museum of Nature Hamburg, Germany (ZMH). The left palp of *Fonteferrea minutissima* was dissected for accurate observation (the right palp is missing, as reported in the original species description).

Initial observation and dissection of the holotype specimen was performed on a Leica M125 stereomicroscope. High-resolution images of the type specimen of *F. minutissima* were taken at the Zoological Institute and Museum Greifswald (Germany) with the BK PLUS Lab system (Dun Inc.) using a Canon 65 mm macro lens mounted on a Canon 5D Mark II camera. Image stacks were captured with Adobe Lightroom and processed using Zerene Stacker under PMax value. Obtained extended focus images were edited using Adobe Photoshop CS 6. Palp was observed and images were taken after submerging the palp in Eugenol (clove oil), with the BK PLUS Lab system, using a 10× objective mounted on a Canon 7D Mark II camera. Raw images were processed as above.

Non-destructive scanning electron microscopy (SEM) of both *F. minutissima* and the palp of *E. minuscula* were performed at the Museum of Nature Hamburg. Specimens were prepared for SEM imaging by temporary dehydration using ethanol solutions from 70% to 100% and then transferred to Hexamethyldisilazane (HMDS 99%) for 3 h. Specimens were mounted on a SEM stub and imaged using a Hitachi tabletop Microscope TM4000 plus. The specimens were not coated and were promptly returned to their original vials containing 70% ethanol immediately after SEM imaging. This procedure ensures their continued preservation, which is crucial for safeguarding type material from destruction and thereby maintaining its availability for future research.

## Results

### Fonteferrea minutissima

Taxon classificationAnimaliaAraneaeFonteferreidae

Wunderlich, 2023

F8F0D24F-5B09-5D63-A87A-499F5A19B7F0

Figs 1, 2, 3, 4, 5, 6, 7, 8, 9, 10, 11, 12, 13, 14, 15, 16

#### Material examined.

***Holotype***: Portugal • ♂; N of Sao Bras de Alportel, Fonteferrea; J. Wunderlich leg.; R244/CJW; ZMH-ARA-A00020712.

#### Description.

**Figures 1–4.**
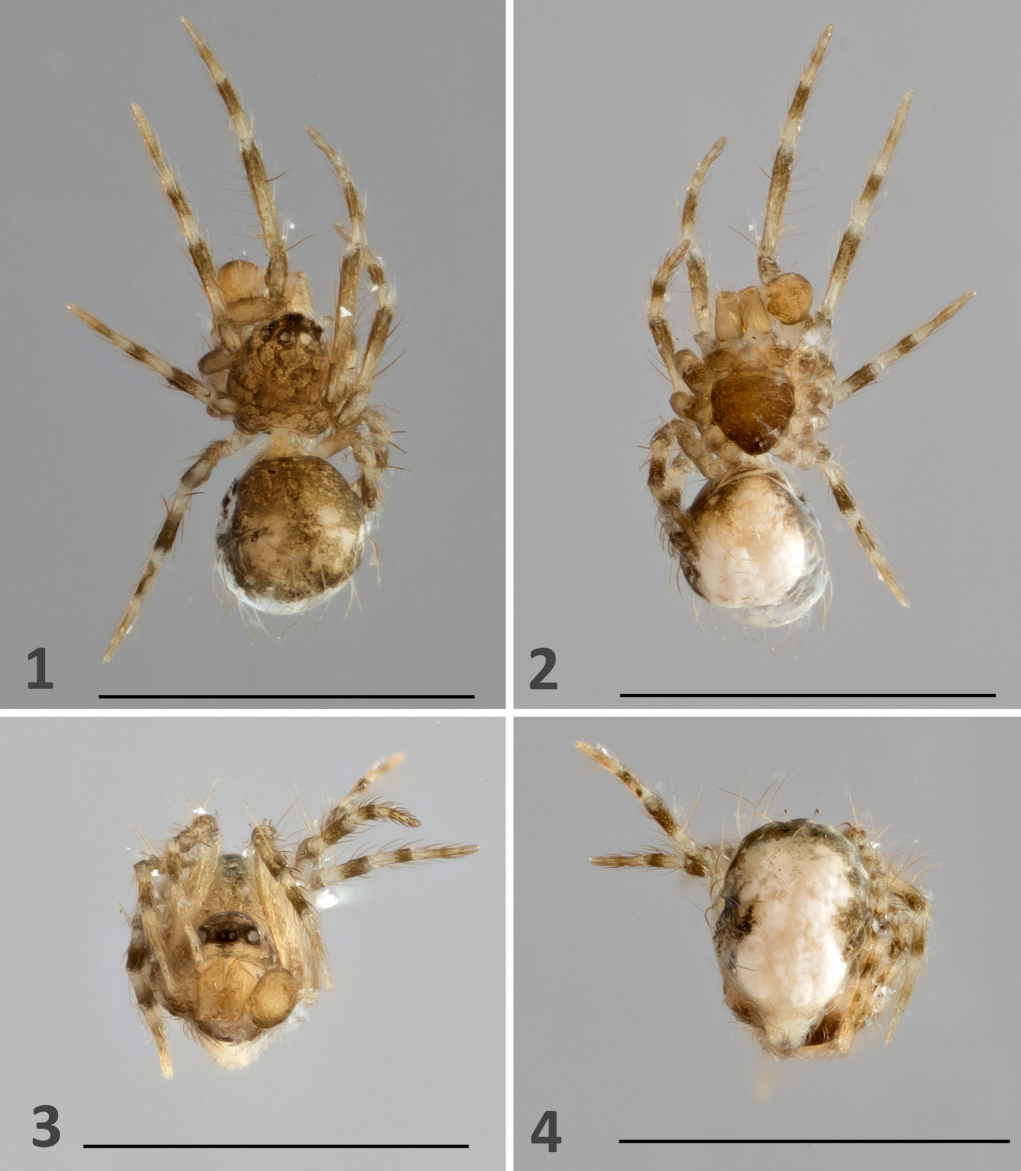
*Fonteferrea minutissima* Wunderlich, 2023. Holotype (ZMH-ARA-A00020712). **1**. Habitus; **2**. Ventral view; **3**. Frontal view; **4**. Posterior view. Scale bars: 1 mm.

**Figures 5, 6.**
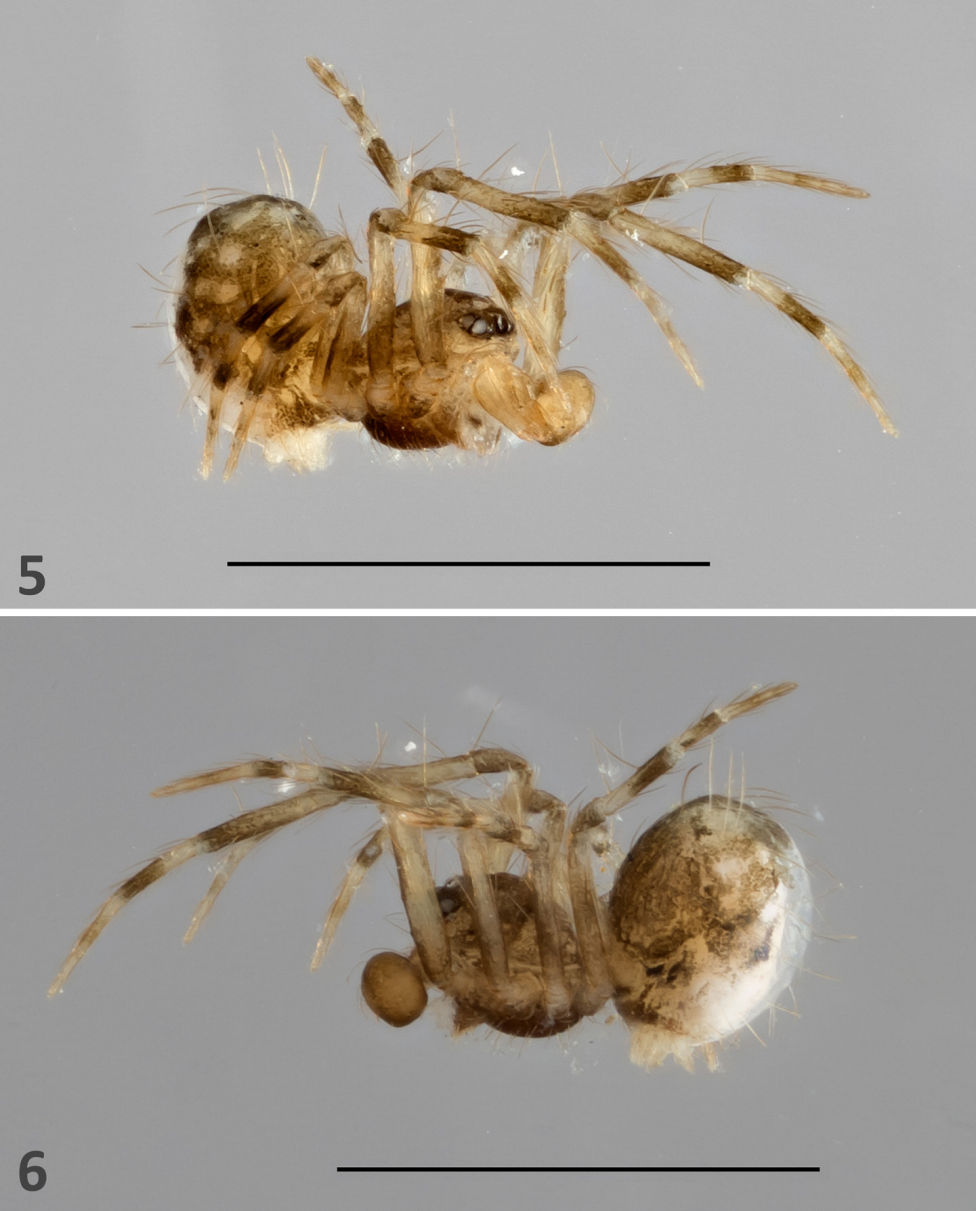
*Fonteferrea minutissima* Wunderlich, 2023. Holotype (ZMH-ARA-A00020712). **5**. Fronto-lateral view; **6**. Lateral view. Scale bars: 1 mm.

**Figure 7.**
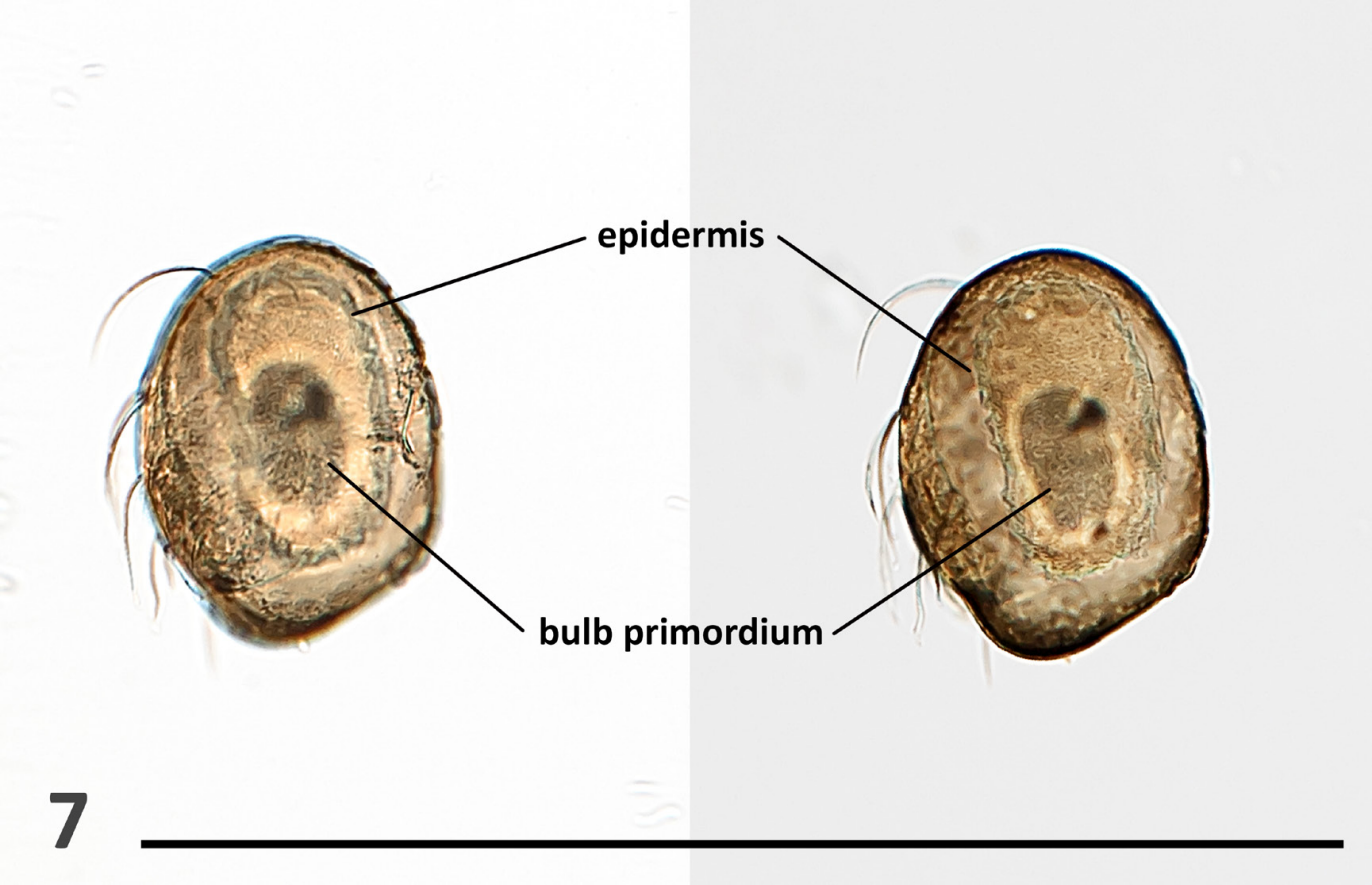
*Fonteferrea minutissima* Wunderlich, 2023. Holotype (ZMH-ARA-A00020712), dissected left bulb, depicted under two different focal planes. Scale bar: 0.5 mm.

**Figures 8, 9.**
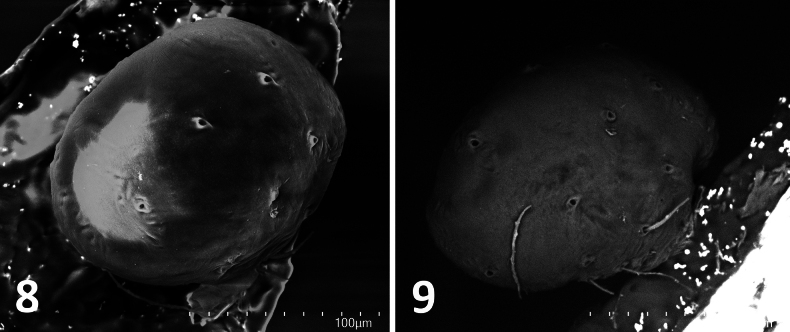
*Fonteferrea minutissima* Wunderlich, 2023. Holotype (ZMH-ARA-A00020712), left bulb. **8**. Dorsal view. **9**. Lateral view. Scale bars: 100 mm.

***Palp***. High-resolution light microscopy and images (Figs 1–3, 5–9) confirm that the palp of the holotype of *F. minutissima* is not “simple,” as originally described, but rather undeveloped and lacking external reproductive structures, features consistent with a subadult male. The undeveloped palp is covered in setae and exhibits internal components characteristic of a developing male palp (Fig. 7; see Quade et al. 2019). These internal structures are visible only under backlight illumination using a compound microscope. In contrast, under stereomicroscopy, the palp appears completely transparent and no structures are discernible, again consistent with observations of subadult males.

Consistent with its subadult stage, the palp of *F. minutissima* lacks an embolus or any other external reproductive structures. Habitus images taken prior to dissection show no evidence of a sclerotized palpal structure (e.g. an embolus; Figs 1–3, 5, 6). The palp, otherwise undamaged, is covered only with setae and shows no indication of a break point or a missing embolus.

***Somatic morphology***. Due to its extremely small size, the somatic morphology of the holotype is difficult to discern, even under high-resolution microscopy and SEM. As the male palpal features are not available since the specimen is a subadult (see above), a thorough evaluation of the somatic morphological traits is essential to assess the taxonomic status of Fonteferreidae as a distinct family (features described by Wunderlich 2023 in italics):

***Raised prosoma***. The prosoma is not conspicuously elevated (Figs 3, 5, 6).

**Figures 10–13.**
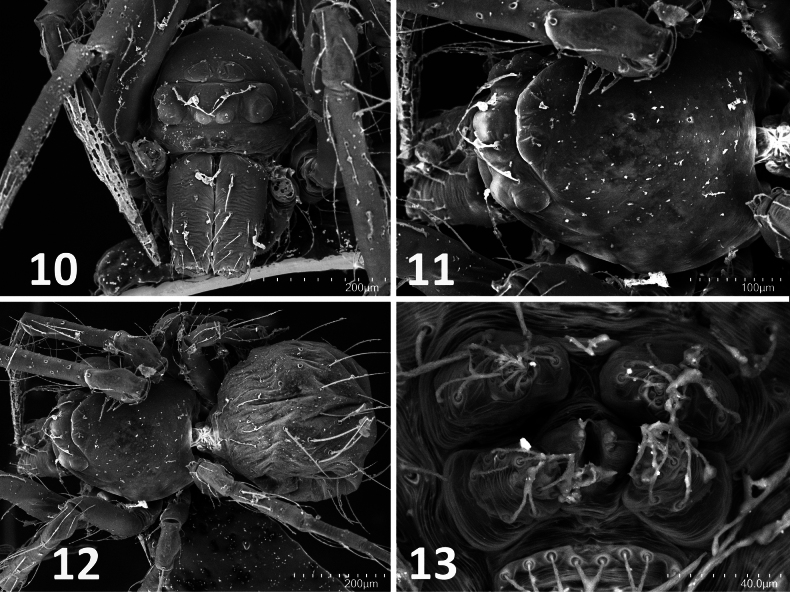
*Fonteferrea minutissima* Wunderlich, 2023. Holotype (ZMH-ARA-A00020712). **10**. Carapace, frontal view; **11**. Carapace, dorsal view; **12**. Habitus, dorsal view; **13**. Spinnerets. Scale bars: 200 mm (**10, 12**); 100 μm (**11**), 40 mm (**13**).

***Short clypeus***. The clypeus is short (i.e. the anterior median eyes are relatively close to the clypeal margin; Fig. 10).

***Opisthosoma quite high, higher than long***. This feature refers to the relative position of the pedicel and the resulting orientation of the abdomen, in which the spinnerets are directed ventrally (Figs 4, 6).

***Eight eyes***. This is a symplesiomorphy in spiders.

***Bristles on legs***. Such “bristles” (macrosetae) are present across all legs and on the abdomen.

***Legs distinctly annulated***. The legs exhibit an annulated coloration pattern (e.g. Fig. 5).

***Body length*** 0.75 mm. Our measurements indicate a body length of 0.81 mm.

In addition to the allegedly diagnostic characters mentioned above, several other features were proposed in support of Fonteferreidae, mainly to justify its exclusion from closely related families (Wunderlich 2023). Among these features, we confirm the absence of sternal pits, clasping spines, cheliceral stridulatory organs, a constriction between tarsi and metatarsi, and a cheliceral keel. The remaining diagnostic features, including the absence of lateral tibial bristles, the absence of long trichobothria on femora and tibiae III–IV, and the absence of a tarsal comb on leg IV, could not be evaluated due to the minute size of the specimen, the use of a non-destructive SEM protocol (without metal coating), and the presence of debris in the specimen obscuring morphological structures.

### Enoplognatha minuscula

Taxon classificationAnimaliaAraneaeTheridiidae

Wunderlich, 2023

F02CA4CB-0B0F-5C5C-B137-C22D4E1F4931

Figs 17, 18

#### Material examined.

***Holotype***: Portugal • ♂; J. Wunderlich leg.; R224/CJW; ZMH-ARA-A00020713.

#### Description.

***Palp***. Our SEM images of the holotype palp (Figs 17, 18) clearly show that the setae in question originate from the cymbium, not from the subtegulum or tegulum.

## Discussion

The empirical value of morphological variation in the adult palps of Araneae for circumscribing species and higher taxa is so well established and robustly supported that the use of this line of evidence is rarely discussed or questioned in most modern taxonomic papers, except in the cases of cryptic species. Detailed descriptions and illustrations of adult male palpal morphology are the standard taxonomic practice in species descriptions of spiders, and thus it is universally agreed that juvenile specimens are insufficient to properly describe and diagnose new species (and consequently new higher taxa). Furthermore, except in cases with unique somatic morphologies or coloration, without the morphological data provided by the genitalia it is not possible in most cases to accurately state whether a specimen belongs to a new species or if it has already been described under another species concept.

Our examination of the holotype palp of *Fonteferrea minutissima* conclusively indicates that the specimen is a subadult male. As a result, any diagnostic features proposed for Fonteferreidae, *Fonteferrea* and *F. minutissima* based on the allegedly adult male palpal morphology should be disregarded since the palpal structures are undeveloped in the holotype.

Additional diagnostic features related to somatic morphology have either not been properly/explicitly defined, are symplesiomorphic, and/or are not unique to Fonteferreidae. For example, the prosoma is not conspicuously elevated compared to other araneoid groups (e.g. in Linyphiidae; Figs 3, 5, 6) nor an objective definition of the term “raised” was provided. Among symphytognathoids, an elevated carapace typically exceeds half the carapace length in height, especially in taxa exhibiting carapace height dimorphism (e.g. in Anapidae, Mysmenidae, and Theridiidae) (see Lopardo and Hormiga 2015: 741, characters 37 and 38). A non-elevated (unraised) prosoma is, as in this case, considered a plesiomorphic condition within spiders. As with the prosoma, no objective definition of the term “short” was provided for the clypeus morphology. Moreover, a short clypeus is a common plesiomorphic trait among spiders and therefore not a reliable diagnostic feature (pers. obs.). The abdominal orientation observed in Fonteferreidae is also common across symphytognathoids and in Theridiidae (see Lopardo and Hormiga 2015: 737, character 5). The symplesiomorphic eight-eye condition does not serve as a diagnostic feature in spiders. A widespread coverage of thick setae (macrosetae) is commonly observed in juveniles and subadults (particularly within symphytognathoids, also theridiids; per. obs.). Furthermore, abdominal “bristles” are also found in Symphytognathidae, Theridiidae, Mysmenidae, and other araneoids (Lopardo and Hormiga 2015), further undermining their diagnostic value. Annulated legs are common across araneomorphs, including the araneoid families Symphytognathidae, Mysmenidae, Theridiidae, Linyphiidae, and Pimoidae (e.g. Hormiga 2008; Lopardo and Michalik 2013; Lopardo and Hormiga 2015; also pers. obs.), and thus it cannot be considered diagnostic. Lastly, as with the opisthosomal orientation and pedicel position, minute adult body size (i.e. less than 2 mm) is a widespread characteristic among symphytognathoids, Theridiidae, and other araneoid groups (see Lopardo and Hormiga 2015: 741, character 34), and therefore small size lacks diagnostic utility.

**Figures 14–16.**
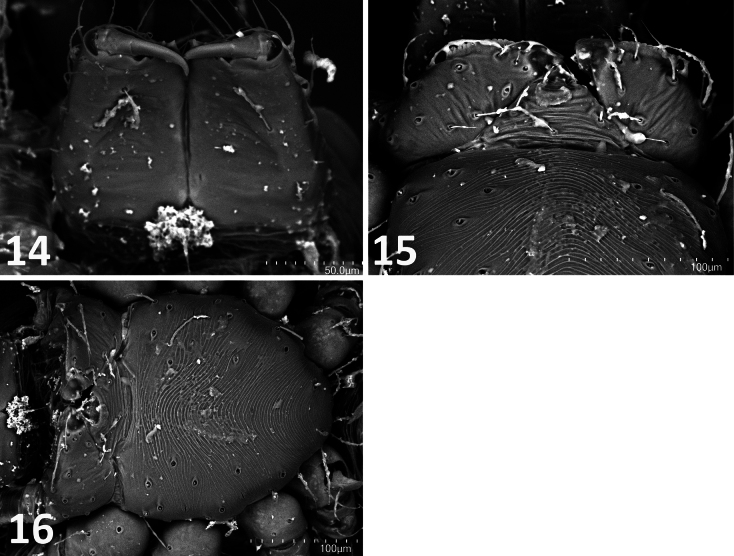
*Fonteferrea minutissima* Wunderlich, 2023. Holotype (ZMH-ARA-A00020712). **14**. Chelicerae, posterior view; **15**. Labium and anterior sternum, ventral view; **16**. Sternum, ventral view. Scale bars: 50 mm (**14**), 100 mm (**15, 16**).

**Figures 17, 18.**
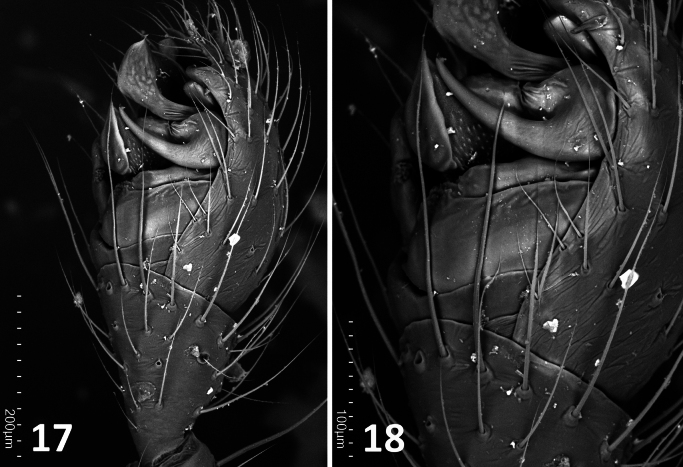
*Enoplognatha minuscula* Wunderlich, 2023. Holotype male (ZMH-ARA-A00020713). **17**. Palp, retrolateral view; **18**. Detail of palp, retrolateral view. Scale bars: 100 mm (**17**); 200 mm (**18**).

Consequently, our examination of the somatic morphology reveals that none of features previously suggested as diagnostic for this putative family, individually or in combination, provide a distinct or coherent set of characters sufficient to justify recognition at the family rank. The presence or position of a few leg macrosetae in a single specimen, particularly in the absence of both a comprehensive comparative assessment across related families and of any phylogenetic justification, is insufficient to support a family taxon or a new species or genus.

In absence of the necessary empirical evidence to support a family rank we recommend that Fonteferreidae is rejected as a valid spider family.

### Taxonomic assessment of *Fonteferrea minutissima*

Given its minute size and subadult developmental stage, the familial placement of the only known specimen of *F. minutissima* is challenging and must rely exclusively on somatic characteristics. To address this, we followed the most comprehensive morphological studies on symphytognathoids to date (Lopardo et al. 2011; Lopardo and Hormiga 2015 and references therein; see also Kulkarni et al. 2023), evaluating only those synapomorphies related to somatic morphology that were accessible to observation in the holotype. Consequently, features associated with sexual dimorphism, such as the metatarsal clasping spine (present only in adult males) and reproductive structures in both sexes, must be logically excluded from consideration.

#### Mysmenidae

In overall appearance *F. minutissima* resembles typical mysmenines (Mysmenidae Simon, 1922). However, it lacks several diagnostic features: the femoral spot (sometimes visible even in subadults; Figs 2, 3, 5), a distinctly thick distal promarginal curved seta on the chelicerae (Fig. 10), and anterior median eyes positioned on a protruded area of the carapace (best observed in SEM and frontal view; Figs 10, 11) (Lopardo et al. 2011: 317; Lopardo and Hormiga 2015: 712–713).

#### Symphytognathidae

*Fonteferrea minutissima* shares with Symphytognathidae Hickman, 1931 an abdominal cuticle with a fingerprint-like pattern covered by distinctly long, thick macrosetae (Fig. 12), as well as the absence of a cheliceral distal promarginal curved seta (Fig. 10). However, it differs in several key features: the presence of a colulus (Fig. 13), the presence of anterior median eyes (Fig. 10), the absence of long trichobothria on tibiae III–IV, and unfused chelicerae along the midline (Figs 10, 14) (Lopardo et al. 2011: 316).

#### Anapidae

The only trait that *F. minutissima* shares with Anapidae Simon, 1895 is the presence of minute anterior median eyes (Fig. 10). It can be distinguished from anapids by its labium with non-concave labial apex and that is not fused to the sternum (Fig. 15), and by the absence of pore-bearing depressions on the prosoma (Lopardo et al. 2011: 316).

#### Theridiosomatidae

*Fonteferrea minutissima* differs from Theridiosomatidae Simon, 1881 by the absence of sternal pits (Figs 15, 16), the absence of long trichobothria on tibiae III–IV, the absence of a cheliceral distal promarginal curved seta (Fig. 10), and anterior median eyes not on a protruding carapace region (Fig. 10) (Lopardo et al. 2011: 315).

#### Synaphridae

*Fonteferrea minutissima* resembles Synaphridae Wunderlich, 1986 in lacking a cheliceral distal promarginal curved seta (Fig. 10), lacking retrolateral distal cheliceral setae (Fig. 14), and having small anterior median eyes (Fig. 10). However, it differs by possessing a narrow, posteriorly located spiracular opening (Fig. 13), the presence of tibial dorsal macrosetae, an unconstricted metatarsus–tarsus joint, and the absence of a cheliceral keel ending in a single promarginal tooth (Fig. 10) (Lopardo et al. 2011: 316).

#### Anterior Tracheal System (ANTS) clade

The ANTS clade, a lineage including all symphytognathoids except the basal Theridiosomatidae (Lopardo et al. 2011: 314; see also Kulkarni et al. 2023), is defined in part by the presence of a row of plumose curved setae on the retromarginal distal margin of chelicerae—a trait absent in *F. minutissima* (Fig. 14).

#### Symphytognathoids and Theridiidae

The overall morphology of symphytognathoids resembles that of small-bodied Theridiidae (e.g. *Steatoda*). Features shared between these taxa and *F. minutissima* include a domed sternum in lateral view (Figs 5, 6), a posterior sternal margin that is neither pointed nor truncate (i.e. “intermediate”; Fig. 16), absence of carapace fovea, minute adult body size (less than 2 mm), a colulus with three or fewer setae (Fig. 13), the presence of abdominal “bristles”, distinctly annulated legs, and the relative position of the pedicel on the abdomen (Lopardo et al. 2011: 313–314; Levi and Levi 1962). As noted above, because of the specimen extremely small size, the use of a non-destructive SEM protocol, and the presence of debris obscuring key features, neither light microscopy nor SEM imaging makes it possible to assess whether *F. minutissima* possesses the tarsal comb on leg IV, or the enlarged PLS aggregate spigots characteristic of theridiids (Griswold et al. 1998). Because theridiids lack femoral macrosetae (Wunderlich 1986; Griswold et al. 1998; Agnarsson 2004), the presence of these structures in *F. minutissima* suggests that the species is not a member of Theridiidae.

In absence of adult specimens and of additional conspecific specimens suitable for DNA sequencing the taxonomic placement of *F. minutissima* remains uncertain, with potential affinities either within symphytognathoids or, less likely based on the presence of femoral macrosetae, Theridiidae. However, one additional morphological trait, the relative length of metatarsus and tarsus, suggests a potential affinity with symphytognathoids. Although considered a continuous character within spiders, metatarsi are typically longer than tarsi in most taxa. In contrast, in both symphytognathoids and *F. minutissima*, the metatarsi are subequal to or shorter than the tarsi (Lopardo and Hormiga 2015).

Due to the limitations of the holotype (a juvenile specimen) for providing proper diagnostic characters, and to the absence of clear synapomorphies supporting a familial assignment, we are unable to determine the taxonomic identity of *F. minutissima*. Accordingly, we designate *Fonteferrea minutissima* as a ***nomen dubium*** within Araneae, and consequently the genus *Fonteferrea* and the family Fonteferreidae are also ***nomina dubia***. If adult specimens of this species are made available, preferably from the type locality, the International Commission of Zoological Nomenclature should be requested to set aside under its plenary power (Article 81) the existing unidentifiable holotype so that a neotype could be designated (ICZN, Article 75.5).

#### Absence of setae on male palpal bulbs

The male copulatory organ of spiders is on the pedipalp and consists primarily of two components: the cymbium and the bulb. The cymbium is the modified, compacted tarsus, while the bulb comprises a set of interrelated sclerites that originate during development within the palpal tarsus, specifically from the bulb primordium. This primordium forms directly beneath the base of the tarsal claw and appears to derive from the same cellular group that gives rise to the claw fundament. Based on this developmental origin, it has been proposed that the bulb sclerites represent an extremely modified form of the adult male pedipalpal claw (Quade et al. 2019). As such, these sclerites lack setae—a trait consistent with their derivation from claw tissue. In contrast, the cymbium, as a remnant of the tarsus, typically bears setae across its surface.

Wunderlich (2023, 2024) noted the presence of “hairs” on the male palpal bulb—distinct from the always setose cymbium—as a “quite rare character”, observed in six spider species across six families. These include three species described by Wunderlich from fossil material (†*Eogamasomorpha rostratis* Wunderlich, 2020 – Tetrablemmidae; †*Palaeophantes paracymbium* Wunderlich, 2023 – Linyphiidae; and †*Paramiagrammopes dexter* Wunderlich, 2021 – Uloboridae) and three extant species, also described by Wunderlich (*Enoplognatha minuscula* Wunderlich, 2023 – Theridiidae; *Fonteferrea minutissima* Wunderlich, 2023 – Fonteferreidae; and *Zelotes lapicidinae* Wunderlich, 2024 – Gnaphosidae). However, Wunderlich (2023: 16) acknowledged that “the exact insertion, the fine structure, and the function of these hairs are still unknown.” We are not aware of any other published accounts documenting setae on the palpal bulb. Closer inspection has cast doubt on these observations in at least two of the extant species. In the case of *F. minutissima*, the palp is covered in setae, but this is expected, as it represents an immature structure still undergoing development and logically lacking any reproductive elements (Figs 7, 8, 9). In the original description of *E. minuscula*, Wunderlich (2023: 30) stated that both the subtegulum and the tegulum of the male palp “bear retrolaterally some shorter and longer thin hairs, originating on small sockets”. However, detailed examination confirms that the setae arise, as predicted, from the cymbium, not from the bulb sclerites (Figs 17, 18).

Given the developmental origin of the bulb sclerites, the minute body size of the species involved (all under 3 mm), and the taphonomic limitations inherent in fossil material, reports of setae on the male palpal bulb might stem from observational errors rather than representing true morphological features. As such, the presence of setae on the bulb should not be considered a reliable diagnostic character until proven otherwise.

N.B. Shortly after having received the peer-reviews of the original submission of our manuscript, a publication by Marc and Déjean (2025) described a putative new species of *Fonteferrea* from southern France. A thorough evaluation of their paper falls beyond the scope of our study since it was published when our work had been completed, submitted for publication, and revised. Nonetheless, we offer a few comments on the findings of Marc and Déjean (2025). These authors examined multiple specimens of both sexes of the putative new species, *F. estageli* Marc & Déjean, 2025. All examined male specimens (11) had only one palp, the left one. The simple, smooth bulb is covered with sparse, long setae and has a fleshy, slightly sinuous micro-depression that appears to contain an indistinct embolus (“cette “structure charnue”, ici légèrement sinueuse, accueille ce qui doit s’apparenter à l’embolus, indistinct”). In the published images the alleged embolus is indeed indistinct, and we fail to see anything that would suggest the presence of an embolus. Their description and images suggest that the studied male specimens are subadults. In the case of the female specimens the authors candidly admit that although no genitalic structures were found, the internal genital structures are present but invisible: “Plusieurs individus a priori adultes ont été disséqués, mais n’ont pas permis d’observer de structures génitales internes; on les considère malgré tout présentes mais donc “non visibles”.” As in the case of the males, we believe that the female specimens are not adults. Intriguingly, some female specimens have been found on debri-covered eggsacs with about 10 eggs. In the theridiid genera *Tidarren* and *Echinotheridion* the males relatively quickly self-amputate after the penultimate molt without exception, and thus subadult males are already one-palped (Knoflach and van Harten 2006). In summary, the study by Marc and Déjean (2025) provides no compelling evidence that the specimens described are adults. Further studies are needed with appropriate methods, such as scanning electron microscopy and sequencing of multiple loci, to elucidate the systematic position of *F. estageli*.

## Conclusions

The description of *Fonteferrea minutissima* as a new genus and species and the erection of the family Fonteferreidae is grounded solely on a single ontogenetically immature specimen lacking definitive morphological autapomorphies and consequently these taxonomic concepts are not supported by an adequate empirical basis. While the features of this juvenile specimen are consistent with the overall morphology of araneoids, we cannot assess the two most widespread synapomorphies of that clade, the paracymbium and the PLS triplet (Hormiga and Griswold 2014). The use of juvenile spider specimens as types contravenes the fundamental principles of zoological nomenclature as outlined by the International Code of Zoological Nomenclature (ICZN 1999), which emphasizes the importance of diagnostic clarity for stability. Using juveniles as type specimens is warranted only under extraordinary circumstances, which are lacking in the case of Fonteferreidae. By failing to adhere to these universally accepted standards, the description of *F. minutissima* lacks the rigor required to justify its recognition as a valid family, genus, and species. Our study of the holotype specimen supports the rejection of Fonteferreidae, of its type genus, and of the type species as valid taxa. In absence of clear, adult-based diagnostic characters and given the unresolved familial placement, we designate *Fonteferrea minutissima*, the genus *Fonteferrea*, and the family Fonteferreidae as ***nomina dubia*** within Araneae. Resolving the taxonomic identity of the holotype specimen of *F. minutissima* will require studying adult material from the type locality.

## Supplementary Material

XML Treatment for Fonteferrea minutissima

XML Treatment for Enoplognatha minuscula

## Additional information

### Use of AI

No use of AI was reported.

### Author ORCIDs

Lara Lopardo https://orcid.org/0000-0003-3481-980X

Nadine Dupérré https://orcid.org/0000-0003-2195-878X

Gustavo Hormiga https://orcid.org/0000-0002-0046-1822

Peter Michalik https://orcid.org/0000-0003-2459-9153

### Data availability

All of the data that support the findings of this study are available in the main text.
